# α6β1- and αV-integrins are required for long-term self-renewal of murine embryonic stem cells in the absence of LIF

**DOI:** 10.1186/s12860-015-0051-y

**Published:** 2015-02-27

**Authors:** Sandhanakrishnan Cattavarayane, Riitta Palovuori, Jayendrakishore Tanjore Ramanathan, Aki Manninen

**Affiliations:** Biocenter Oulu, Oulu Center for Cell-Matrix Research, Faculty of Biochemistry and Molecular Medicine, University of Oulu, Aapistie 5, Oulu, 90220 Finland; Current address: Université de Lorraine, CS 25233, Nancy, cedex, 54052 France

**Keywords:** Embryonic stem cells, Integrin, Extracellular matrix, Self-renewal, Adhesion

## Abstract

**Background:**

The growth properties and self-renewal capacity of embryonic stem (ES) cells are regulated by their immediate microenvironment such as the extracellular matrix (ECM). Integrins, a central family of cellular ECM receptors, have been implicated in these processes but their specific role in ES cell self-renewal remains unclear.

**Results:**

Here we have studied the effects of different ECM substrates and integrins in mouse ES cells in the absence of Leukemia Inhibitory Factor (LIF) using short-term assays as well as long-term cultures. Removal of LIF from ES cell culture medium induced morphological differentiation of ES cells into polarized epistem cell-like cells. These cells maintained epithelial morphology and expression of key stemness markers for at least 10 passages in the absence of LIF when cultured on laminin, fibronectin or collagen IV substrates. The specific functional roles of α6-, αV- and β1-integrin subunits were dissected using stable lentivirus-mediated RNAi methodology. β1-integrins were required for ES cell survival in long-term cultures and for the maintenance of stem cell marker expression. Inhibition of α6-integrin expression compromised self-renewal on collagen while αV-integrins were required for robust ES cell adhesion on laminin. Analysis of the stemness marker expression revealed subtle differences between α6- and αV-depleted ES cells but the expression of both was required for optimal self-renewal in long-term ES cell cultures.

**Conclusions:**

In the absence of LIF, long-term ES cell cultures adapt an epistem cell-like epithelial phenotype and retain the expression of multiple stem cell markers. Long-term maintenance of such self-renewing cultures depends on the expression of β1-, α6- and αV-integrins.

**Electronic supplementary material:**

The online version of this article (doi:10.1186/s12860-015-0051-y) contains supplementary material, which is available to authorized users.

## Background

Embryonic stem (ES) cells are isolated from the inner cell mass (ICM) of the blastocyst. Similar to ICM cells, ES cells are pluripotent and can self-renew thereby making them invaluable tools in the field of regenerative medicine. During embryonic development ICM differentiates into epiblast cells and subsequently to all the different cell types of the adult via several morphological transformations involving sequential epithelial-to-mesenchymal (EMT) and mesenchymal-to-epithelial transitions (MET) [1]. In the developing embryo, dynamic changes in the surrounding extracellular matrix (ECM) regulate the fates of embryonic cells thereby participating to the complex orchestration of the body plan [2]. In particular, the laminin-rich basement membrane has been shown to be an essential regulator of cellular differentiation during embryonic development [3]. Despite significant advances in the characterization of cell-ECM interactions in differentiating ES cells, the molecular machineries underlying ECM-mediated regulation of stem cell properties remain incompletely understood.

Integrins are the major cellular ECM-receptors that have also important signaling functions [4,5]. ES cells express a remarkable repertoire of integrins including several laminin-, fibronectin- and collagen-binding integrin heterodimers [6,7]. Tryggvason and coworkers reported that laminin-511 substrate allowed prolonged culture of pluripotent murine and human ES cells *in vitro* in the absence of leukemia inhibitory factor (LIF) that is generally required to maintain ES cells in undiffentiated state in feeder cell-free *in vitro* cultures [6,8,9]. ES cells adhered to LN-511 mainly via α6β1- and αVβ1-integrins and not only retained expression of pluripotency markers but also the capacity to contribute to chimeric tissues when injected into mouse blastocysts. On the contrary, another study on murine ES cells reported that integrin-mediated binding to laminin, fibronectin or collagen activated a signaling cascade leading to suppression of ES cell self-renewal [7]. Recently, the Hubbell laboratory developed and tested various synthetic substrates for their capacity to maintain mouse ES cell self-renewal and concluded that simultaneous ligation of α5β1-, αVβ5-, α9β1- and α6β1-integrins promotes stemness of ES cells [10]. These integrins have also been implicated in the regulation of mouse and human ES cell self-renewal in a number of other studies performed under various growth conditions [11-14]. Finally, Suh and Han found that α2β1-integrin promoted ES cell self-renewal on collagen substrate [15]. Integrin-mediated cell-ECM interactions are thus clearly involved in the regulation of stem cell properties although the specific role(s) of integrins whether they promote or inhibit self-renewal remains unclear.

Here we have addressed the functional roles of cell-matrix interactions on ES cell differentiation and self-renewal by studying the effects of selected ECM substrates in combination with RNAi-mediated silencing of integrin expression. To focus our studies on the role of the ECM we performed all experiments in feeder-free culture conditions in the absence of LIF. Upon acute LIF withdrawal ES cells adopted cobblestone morphology and displayed transient changes in the expression of key stem cell factors indicative of a transition from the ground-state pluripotent ES cells into so-called primed epistem cell (epiSC)-like cells. Interestingly, these cells could be efficiently propagated for up to ten passages in the absence of LIF on all other substrates except on collagen I (Col-I) to which cells adhered poorly and were often lost during the culture. On all the other substrates prolonged culture led to restoration of an ES cell-like expression profile of stemness markers. α6-integrins were found to be required for self-renewal marker expression on collagen substrate whereas αV-integrins were required to maintain ES cell cultures on LN-511 in the absence of LIF. Inhibition of the expression of β1-integrins that can pair with both α6- and αV-integrins, led to self-renewal defects on all of the substrates studied. These data suggest that α6β1-integrins are crucial for ES cell self-renewal and survival on collagen-rich substrates whereas αV-integrins appear to play a role by regulating adhesive properties and differentiation of ES cells on laminin.

## Results

### The effect of the ECM matrix on the ES cell morphology and adhesion

**Figure 1:**
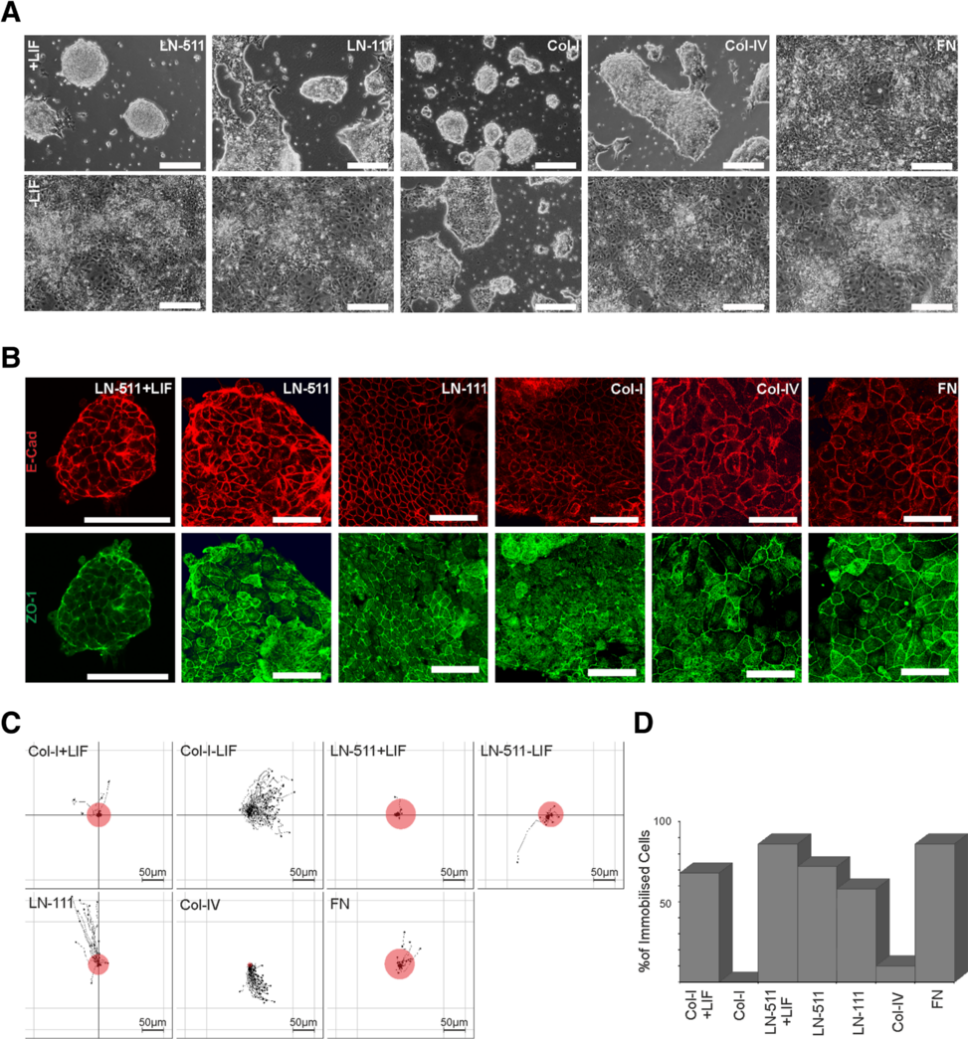
**LIF withdrawal induces a morphological change in ES-D3 cells from multilayered clusters to epithelial monolayers and impairs ES-D3 adhesion to collagen. A)** ES-D3 cells were cultured for 5 days on tissue-culture dishes coated with LN-511, LN-111, Col-I, Col-IV or FN in the presence or absence of 10 ng/ml of LIF and imaged using a phase contrast microscope equipped with a CCD-camera. Scale bar is 100 μm. **B)** ES-D3 cells were cultured on LN-511 in the presence or absence of LIF or on LN-111, Col-I, Col-IV or FN in the absence of LIF, fixed with 4% PFA and stained for an epithelial AJ marker E-Cad (red) and a TJ marker ZO-1 (green). Cells were imaged using confocal microscopy and a single optical slice at the level of TJs (as determined by chicken-wire-like ZO-1 staining) is shown. Scale bar is 50 μm. **C)** ES-D3 cells were counted and seeded (1000 cells/mm^2^) onto tissue culture dishes (3.5 cm Ø) coated with the indicated ECM substrates and individual cells were tracked at 2 minutes intervals upon their contact with the ECM. The amount of adherent (immobile) cells was determined as described in materials and methods. The data shown comes from 2 independent experiments in which at least 50 individual cells were tracked. The relative amount of immobilized cells is depicted with differentially sized red circles the center of the graphs. **D)** A graph showing the percentage of ES-D3 cells immobilized on the indicated substrates within the first 30 minutes upon seeding.

To study the role of the ECM on ES cell self-renewal we seeded ES cells onto tissue culture plates coated with collagen-I (Col-I), Col-IV, laminin-111 (LN-111), LN-511 or fibronectin (FN) in absence of LIF. Initially, we adapted ES-D3 cells into feeder-free cell culture conditions where ES cell pluripotency was maintained by addition of LIF (10 ng/ml) into the culture medium. In the presence of LIF, ES-D3 cells grew as multilayered spherical colonies of tightly packed small cells occasionally surrounded by cell monolayers on all substrates, except on FN where they spread efficiently and formed a monolayer (Figure 1A). In the absence of LIF ES cells on most ECM substrates spread efficiently and formed monolayers composed of cobblestone-like cells. An exception was Col-I on which ES-D3 cells appeared to adhere poorly and where they grew as partially multilayered colonies (Figure 1A).

To analyze cellular morphology and organization in more detail the cells were fixed and stained for epithelial adherens junction (AJ) marker E-cadherin (E-Cad) and tight junction (TJ) marker Zonula Occludens (ZO)-1. The spherical ES cell colonies cultured in the presence of LIF contained E-Cad that was mainly localized to cell-cell boundaries (Figure 1B). ZO-1 displayed a diffuse staining pattern roughly co-localizing with E-Cad. In the absence of LIF, ES-D3 cells displayed robust E-Cad-staining at lateral membranes and accumulation of ZO-1 into sub-apical chicken-wire-like staining pattern indicative of the epithelial nature of the monolayers when grown on laminins, FN or Col-IV. E-cad was seen laterally also in cells grown on Col-I substrate but the sub-apical ZO-1 staining pattern appeared slightly more fragmented than on other substrates (Figure 1B).

Next we examined the adhesive properties of ES cells freshly seeded onto different substrates. Because the spherical multilayered colony morphology in the presence of LIF was best preserved on LN-511 and Col-I-coated dishes, these two substrates were used as undifferentiated controls for subsequent experiments focusing on the LIF-free cultures on the different ECM substrates. ES-D3 cells were seeded onto LN-511, LN-111, Col-I, Col-IV and FN-substrates in the absence of LIF or onto LN-511 and Col-I in the presence of 10 ng/ml LIF and imaged at 2 minutes intervals using automated multipoint timelapse microscopy. Individual cells were tracked and the tracks were analyzed as described in materials and methods. In the presence of LIF, ES cells adhered efficiently to both LN-511 and Col-I as evidenced by rapid immobilization of cells despite the apparent fluid motion due to movement of the motorized stage during imaging (Figure 1C, D; see also Additional file 1: Movie S1). In the absence of LIF, ES cells became rapidly immobilized on LN-511-, FN- and LN-111-coated substrates (Figure 1C, D; Additional file 1: Movie S1). However, on Col-I and Col-IV coated wells ES cells were just hovering on the substrate indicating poor adherence (Figure 1C, D; Additional file 1: Movie S1). Thus, LIF-treated ES cells adhered to all of the substrates tested within the first 60 minutes whereas in the absence of LIF ES cells did adhere to laminins and fibronectin but adhered poorly to collagens within this time early frame.

### Acute LIF-withdrawal induces an epiSC-like expression profile in ES-D3 cells

**Figure 2:**
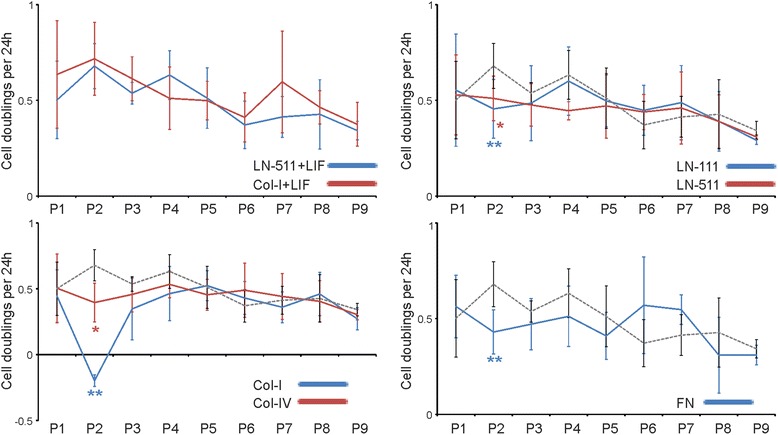
**Long-term cultures of ES-D3 cells can be established in the absence of LIF.** ES-D3 cells were seeded (300 cells/mm^2^) onto LN-511-, LN-111-, Col-I-, Col-IV- or FN-coated tissue culture wells (3.5 cm Ø) and grown in the presence or absence of 10 ng/ml of LIF, as indicated. Upon confluency the cells were trypsinized, counted and reseeded at 300 cells/mm^2^. Cell doubling index was calculated for each time point as average number of divisions by a cell per 24 hours. The graph represents data from 3–4 independent experiments performed in triplicates except for cells grown on Col-I in the absence of LIF where only 2 out of 6 samples contained enough cells to be reseeded after P2 due to cell loss during culture. P-values were calculated using student’s *t*-test and are marked with an asterisk (* < 0.05; ** <0.005).

To examine the effects of the different ECM substrates on self-renewal of ES cells we set up long-term cultures of the ES-D3 cells. Tissue culture wells were coated with LN-511, LN-111, Col-I, Col-IV or FN and 300 ES-D3 cells/mm^2^ were seeded into each well in the absence of LIF. ES cells seeded onto LN-511 or Col-I in the presence of LIF were used as positive controls for self-renewal capacity. Upon reaching ~80% confluency the cells were trypsinized and counted to determine a cell doubling index (a relative measure of cell divisions within 24 h) after which cells were reseeded onto new wells freshly coated with the same substrate from which the cells were harvested. These cultures were kept going as long as sufficient number of cells could be recovered for counting and reseeding. We could consistently establish long-term cultures (up to passage 10; ~2 months) of ES-D3 cells on all substrates except Col-I where cells underwent a proliferative crisis usually within the second passage (Figure 2A). Despite this problem we did occasionally (2 out of 6 samples) obtain enough material to perform further analysis of also the long-term cultures on Col-I substrate (see below).

**Figure 3:**
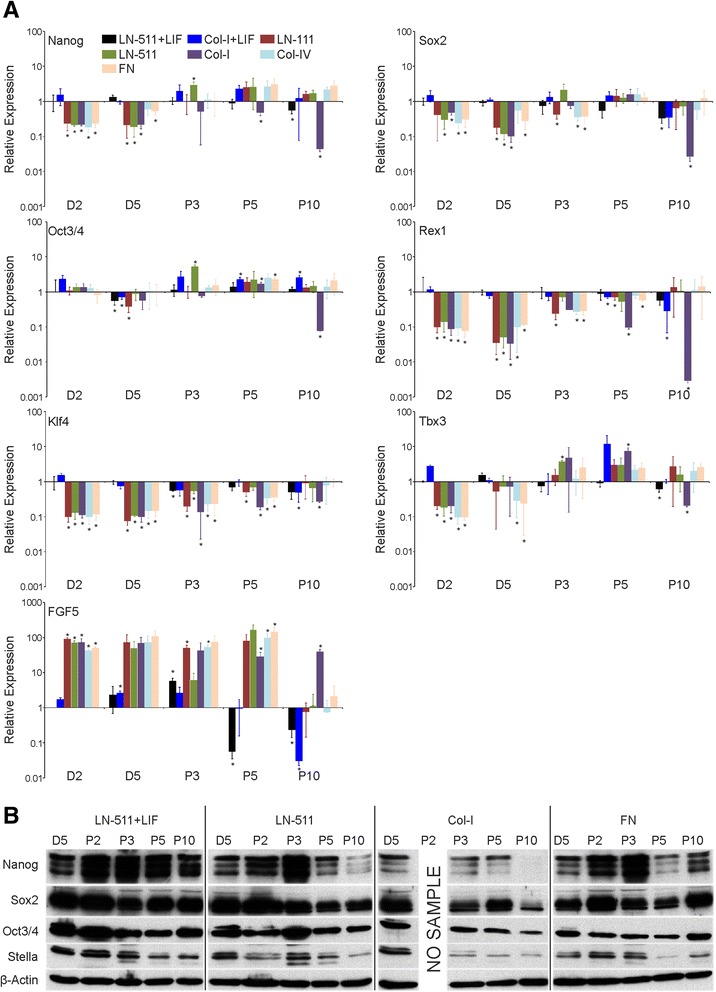
**Prolonged culture of ES-D3 cells in the absence of LIF leads to an enrichment of epithelial self-renewing cells with an ES cell-like transcription profile. A)** ES-D3 cells were cultured as indicated in Figure 2 and total RNA samples were collected at indicated time point. The mRNA expression levels of self-renewal markers (Nanog, Sox2, and Oct3/4), ES cell markers (Rex1, Klf4 and Tbx3) and a differentiation marker (FGF5) were determined using qPCR analysis. The data show means +/−STD of replicates from 2–3 independent samples. P-values (student’s *t*-test) < 0.005 are marked with an asterisk (*). **B)** ES-D3 cells were grown as above and lysed at indicated time points followed by SDS-PAGE and western blot analysis using Nanog, Sox2, Oct3/4 and Stella antibodies to study the protein expression levels of these stem cell markers. β-actin was used as a loading control. The data shown is representative of 2–3 experiments with similar results.

One consistent finding upon LIF withdrawal was that the cell colonies changed their shape from multilayered tightly packed clusters into an epithelial monolayer. ES cells are precursors of another type of self-renewing stem cells, so-called epiSCs that resemble the epiblast cells in early embryos [16-18]. To study the pluripotency and self-renewal properties of the LIF-depleted cells in more detail we next determined the mRNA expression levels of selected self-renewal (Nanog, Sox2 and Oct3/4), stem cell (Rex1, Klf4 and Tbx3) and a differentiation/epiSC (FGF5) markers in the different ES cell cultures using quantitative PCR analysis (qPCR; Figure 3A). LIF-mediated signaling has been reported to activate three signaling cascades, Jak/STAT-, MAPK- and PI3K/Akt-pathways [19]. These pathways synergistically regulate the activities of Klf4 and Tbx3 transcription factors that in turn maintain sufficient expression of the core stem cell factors such as Nanog, Sox2 and Oct 3/4. Reduced expression-1 (Rex1) is another widely used marker for pluripotent stem cells [20]. FGF5 marks the transition from pre- to post-implantation epiblast and is induced during differentiation of ES cells into epiSCs [21]. Again, ES-D3 cells grown on LN-511 or Col-I in the presence of LIF were used as controls and all samples were normalized and compared relative to the LN-511 + LIF sample at day 2. Regardless of the matrix-coating used, the expression of FGF5 was highly induced after LIF-withdrawal, whereas levels of Nanog, Sox2, Klf4, Tbx3 and Rex1 were downregulated suggesting that ES-D3 cells differentiated into epiSC-like cells (Figure 3A). The expression levels of Oct3/4 mRNA were relatively stable. Interestingly, upon prolonged culture the cells showed a gradual shift towards a more ES cell-like mRNA expression profile (Figure 3A). Some markers returned to ES cell like levels during passages 3–5 (Nanog, Tbx1) whereas FGF5 displayed a late response such that downregulation of FGF5 mRNA expression was not yet evident in P5 samples but was prominent in P10 samples (Figure 3A). A notable exception was cells grown on Col-I without LIF in which the expression levels of essentially all of the studied ES cell markers were strongly reduced and FGF5 levels remained high (Figure 3A). Curiously, late passage samples from ES-D3 cells grown on Col-IV in the absence of LIF did maintain the expression of stemness markers and also showed downregulated levels of FGF5 expression.

The protein levels of Nanog, Sox2 and Oct3/4 were studied by western blotting. Despite the observed downregulation of the Nanog and Sox2 mRNAs at early time points, high levels of Nanog and Sox2 protein expression were maintained until approximately third passage in ES cells seeded onto LN-511 or FN substrates (Figure 3A, B). In contrast, Nanog was rapidly downregulated in cells grown on Col-I substrate (Figure 3B). The cell numbers in Col-I substrate in the absence of LIF strongly declined such that all of the remaining cells at passage 2 were used for reseeding and thus no protein sample could be obtained for this time point. In agreement with the mRNA analysis the protein levels of Oct3/4 were relatively stable, although a noticeable reduction was seen in ES cells seeded onto Col-I and FN substrate (Figure 3B). We also studied the protein expression levels of Stella, an ES cell marker that is epigenetically silenced in epiSCs [22]. While there was a tendency to see a drop of Stella expression on all substrates studied, visible levels of Stella protein were retained on all three substrates (Figure 3B). Of note, a decline in the levels of Stella was observed also in late passages of LIF-treated cells (Figure 3B). These data suggest that efficient adhesion correlates with ES cell self-renewal. Since collagen substrates did not efficiently support ES-D3 cell adhesion, LIF-treated cells seeded onto LN-511 were used as a control in all subsequent experiments.

### Integrins mediate the ECM-derived signals to regulate ES cell self-renewal

**Figure 4:**
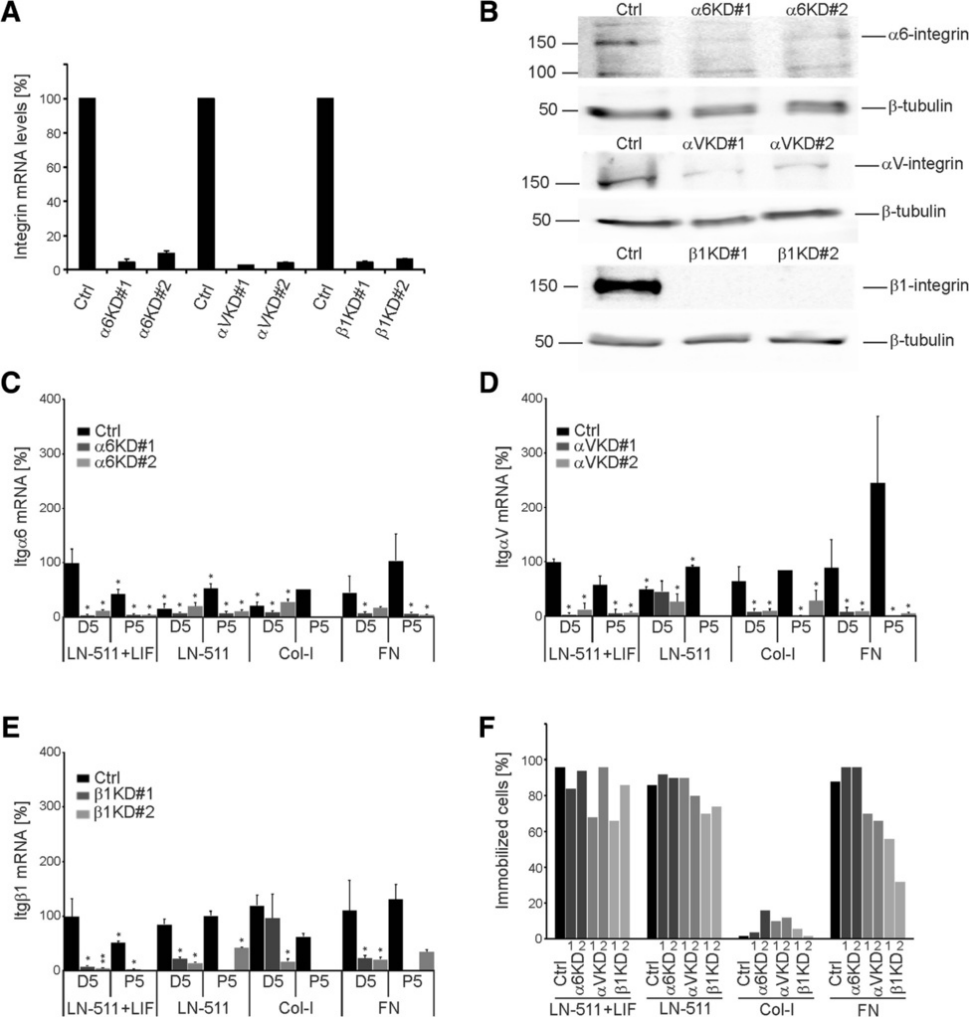
**Characterization of Itgβ1-, ItgαV- and Itgα6-KD ES-D3 cells and their adhesive properties. A)** ES-D3 cells expressing the indicated shRNAs were generated using lentiviral vectors and the efficiency of the target mRNA depletion was determined by qPCR. Data shows means +/− STDs from 3 independent experiments. **B)** Control (Ctrl), Itgα6-knockdown (KD), ItgαV-KD and Itgβ1-KD ES-D3 cells were seeded onto LN-511-coated cells and grown for 3 days in the presence of 10 ng/ml LIF. Cells were lysed and lysates were subjected to SDS-PAGE and western blotting analysis using the indicated integrin antibodies. β-tubulin antibody was used as a loading control. Data shown is representative from 2–3 independent experiments. **C)** Expression of α6-, **D)** αV- and **E)** β1-integrins in ES-D3 cells. ES-D3 cells were seeded onto indicated substrates in the presence or absence of LIF (10 ng/ml). Cells were harvested at day 5 or passage 5, RNA isolated and the expression levels of the mRNAs for the three integrin subunits was determined by qPCR. Data shown is representative of two independent experiments performed in duplicates. **F)** Control (Ctrl), Itgα6-, ItgαV- and Itgβ1-KD ES-D3 cells were trypsinized, counted and seeded (1000 cells/mm^2^) onto tissue culture wells coated with LN-511 (+/− LIF), Col-I or FN as indicated. Adhesion onto different substrates was determined as described in Figure 1C,D.

As integrins are the major class of cellular ECM-receptors we addressed the functional roles of selected integrin subunits in LIF-free ES cell cultures. Extensive characterization of integrin expression profiles in mouse ES cells have been reported [6,7,10; see Additional file 2: Table S1]. By using different approaches and criteria, these studies implicated several β1-integrins, namely α6β1, αVβ1, α5β1, α2β1 and α9β1, in the regulation of ES cell self-renewal. In addition, αVβ5-integrin function has been associated with this process [13]. To study the specific role of selected integrins in the regulation of ES cell self-renewal in the absence of LIF we used lentiviral vectors to stably deplete the expression of β1-, α6-, αV-integrins. Integrin β1-subunit is found in a number of collagen-, laminin- and fibronectin-binding integrin heterodimers and it is essential during early development [23-26]. Out of the many β1-containing integrins, α6β1-heterodimer is highly expressed in ES cells and appears to convey important ECM-mediated signals to regulate ES cells self-renewal in different models [6,10-12,27]. αV-integrin forms heterodimers with a number of different β-subunits and can also mediate β1-integrin-independent binding to FN [28]. ES-D3 cells were infected with concentrated lentiviral vectors to generate β1-, αV- or α6-integrin knockdown (Itg-KD) cell lines. A virus vector containing an empty shRNA expression cassette was used as a control. Two independent shRNA constructs were used for each of the three targets and knockdown efficiencies were confirmed by qPCR (Figure 4A) and western blotting (Figure 4B). The knockdown efficiencies were maintained throughout the duration of the experiments (Figure 4C-E). However, Itgβ1-KD cells did not survive freezing and thawing and thus the role of β1-integrin could only be studied in freshly transduced ES cells.

The adhesive properties of the different Itg-KD ES-D3 cell lines on LN-511, Col-I and FN substrates were studied using the timelapse microscopy-based cell tracking analysis. One of two shRNAs for both β1- and αV-integrins caused moderately reduced adhesion to LN-511 in the presence of LIF but since this effect was not obvious with the second shRNAs these may be off-target effects (Figure 4F). Notably, only moderate adhesion defects (~70% of the Itgβ1-KD cells adhered) were observed in Itgβ1-KD cell populations on LN-511 in the absence of LIF, and Itgα6- and ItgαV-KD cells adhered to LN-511 as well as the controls (~90% of the cells adhered; Figure 4F). As noted earlier, Col-I did not support efficient adhesion for any of the cell lines (Figure 4F). Both Itgβ1- and ItgαV-KD cells had a significant adhesion defect on FN (Figure 4F).

**Figure 5:**
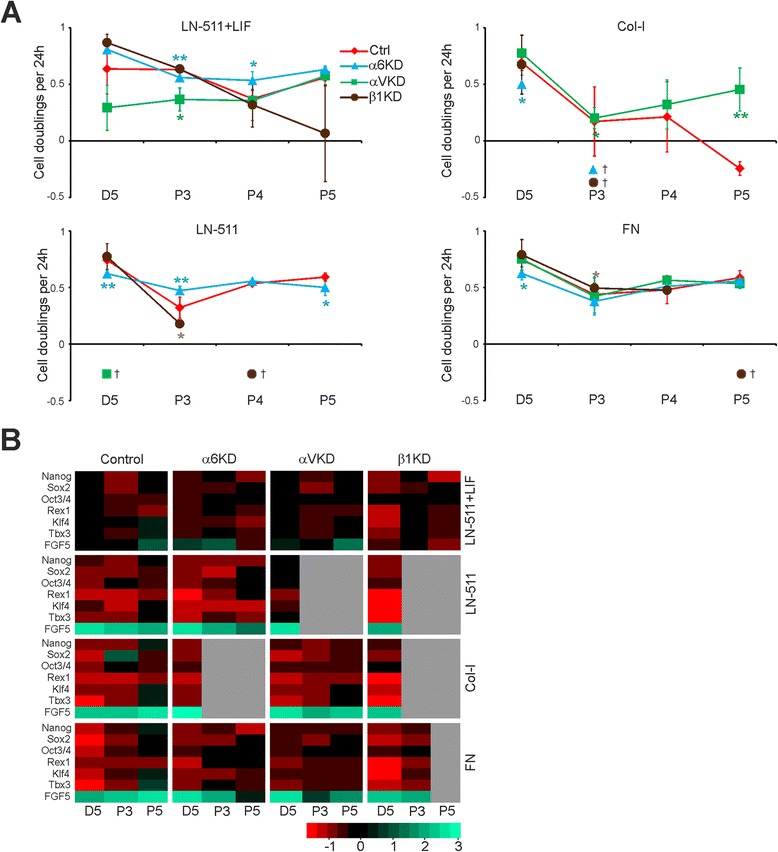
**Integrins are important regulators of the self-renewal capacity of ES-D3 cells. A)** Control (Ctrl), Itgα6-, ItgαV- and Itgβ1-KD ES-D3 cells (300 cells/mm^2^) were seeded onto LN-511-, Col-I- or FN-coated tissue culture dishes (3.5 cm Ø) and cultured in the absence or presence of 10 ng/ml of LIF. Upon confluency the cells were trypsinized, counted and reseeded at 300 cells/mm^2^. Cell doubling index was calculated for each time point as described in materials and methods. The graph represents data from 2 independent experiments performed in duplicates. Cross (†) indicates samples where sufficient amount of cells could not be harvested for analysis. **B)** Control, Itgα6-, ItgαV- and Itgβ1-KD ES-D3 cells were grown on LN-511-, Col-I- and FN-coated surfaces in the absence of LIF, total RNA was extracted at day 5, passage 3 (P3) and P5 and the mRNA expression levels of self-renewal markers (Nanog, Sox2, and Oct3/4), ES cell markers (Rex1, Klf4 and Tbx3) and a differentiation marker (FGF5) were analyzed by qPCR. The data is shown as heat maps where red indicates downregulation of the mRNA and green indicates up-regulation of the respective mRNAs compared to ES-D3 cells grown on LN-511 in the presence of LIF on day 5. The raw data values of means +/−STD are shown in Figure S1 (see Additional file 3). The graph represents data from 2 independent experiments performed in duplicates. Gray color indicates no data due to loss of cells during culture.

To analyze the potential roles of these integrins in ES cell self-renewal, control and the Itg-KD cells were cultured on LN-511, Col-I and FN in the absence of LIF. Cells grown on LN-511 in the presence of LIF were used as controls in each case. The cell doubling index in the different cell lines was determined as described earlier (Figure 2). The control virus-infected cells behaved as wild-type ES-D3 cells and could be efficiently maintained without LIF on LN-511 and FN substrates but not on Col-I (Figure 5A). Itgβ1-KD cells could be passaged up to one month (P5) on LN-511 in the presence of LIF, whereas in the absence of LIF the cultures could not be maintained beyond first two passages (Figure 5A). Itgα6-KD cells could be cultured for several passages in the presence or absence of LIF on LN-511 as well as on FN substrate. Although the cell doubling rates were slightly reduced ItgαV-KD cells could be cultured on LN-511 in the presence of LIF (Figure 5A). In the absence of LIF ItgαV-KD cells appeared to detach as cell colonies grew bigger although individual Itgα V-KD cells adhered as well as the control ES-D3 cells upon contact with LN-511 substrate (Figures 4F, 5A). All cell lines adhered poorly to Col-I and were lost during early passages except for ItgαV-KD cells which despite poor adhesion was the only cell line that consistently survived until passage 5 (Figure 5A). On FN, Itgβ1-KD cultures were lost at later passages while ItgαV- and Itgα6-KD cells could be maintained (Figure 5A). We noted that the cell loss in collagen-seeded Itgα6- and Itgβ1-KD cells and in LN-511 grown ItgαV- and Itgβ1-KD cells coincided with significantly reduced KD-efficiencies in the remaining cells suggesting negative selection against efficient silencing of α6- or β1-integrins on collagen I and αV- or β1-integrins on LN-511 (Figures 5A, 4C-E).

We next studied the stemness marker expression in ES-D3 control and the different Itg-KD cells seeded onto LN-511, Col-I and FN substrates in the absence of LIF. Cells seeded onto LN-511 in the presence of LIF were used as a control. As noted earlier, LIF withdrawal systematically led to downregulation of stem cell marker expression and upregulation of FGF5 in all conditions and cell lines (Figure 5B; see also Additional file 3: Figure S1). This effect was particularly prominent in control cells on FN and in Itgβ1-KD cells on all substrates (Figure 5B). In contrast, ItgαV-KD cells were slightly less responsive to LIF withdrawal when compared with controls on LN-511 and FN. Vast majority of the ItgαV-KD cell colonies detached on LN-511 and not enough samples could be obtained for analysis for later time points. ItgαV-KD cells survived on FN and Col-I, where they did not show major changes in the expression profile of the different markers when compared with controls (Figure 5B). Finally, when comparing the stemness profile of the different Itg-KDs with control cells in the presence of LIF it was found that while ItgαV-KD cells demonstrated a similar stemness marker expression profile as the controls, Itgα6- and Itgβ1-KD cells had reduced expression levels of Nanog, Klf4 and Tbx3 at passage 5. It was also noted that the expression levels of α6-integrin were downregulated upon LIF withdrawal (Figure 4C). These data suggest that α6β1-integrin function is important for the maintenance of pluripotency of ES-D3 cells.

## Discussion

In this study we addressed the role of the extracellular matrix components and selected integrins in the regulation of ES cell self-renewal under feeder-free culture conditions and in the absence of ectopic factors (LIF) that might artificially promote ES cell self-renewal. It was found that on all studied ECM substrates ES cells initially appeared to differentiate towards epiblast-like epiSCs [16-18]. These cells grew as polarized epithelial monolayers and could be maintained in culture for at least ten passages in the absence of LIF on most substrates. Prolonged culture without LIF appeared to enrich for cells which displayed epithelial characteristics (see Additional file 4: Figure S2) and expressed high levels of multiple stemness markers. Col-I substrate was an exception as it did not support strong adhesion or proliferation of ES cells in the absence of LIF. The finding that ES cells adhere poorly on Col-I substrate is in agreement with an earlier report by Hayashi and coworkers [7]. Hayashi et al. concluded that integrin-mediated contact with laminin and FN may negatively affect ES cell self-renewal while lack of integrin activation on collagen substrate would promote ES cell self-renewal [7]. In our study ES cells cultured in the absence of LIF on Col-I did show signs of differentiation (upregulation of FGF5 and downregulation of Nanog, Sox2, Rex1, Klf4 and Tbx3) as did cells grown on laminin or FN where the cells readily adhered. Moreover, upon prolonged culture the levels of ES cell marker mRNAs increased on all other substrates except on Col-I. A critical difference is the use of ectopic inhibition of ES cell differentiation, as we omitted LIF from the culture medium while Hayashi *et al.* kept LIF in their setup [7]. It is possible that addition of even a small amount of LIF is sufficient to override the need for efficient integrin-mediated adhesion. LIF promoted the survival and growth of ES-D3 cells on Col-I whereas in the absence of LIF, cells adhered poorly leading to significant cell loss particularly during the early passages. The remaining cells that did adhere to Col-I in the absence of LIF displayed downregulated levels of multiple stem cells markers. Col-IV substrate was similarly a poor substrate for freshly seeded ES cells but unlike Col-I, Col-IV substrate did support self-renewal in our long-term culture setup. Since levels of I-domain containing collagen-binding integrins are low in mouse ES cells [6,7,10,29], the differential capacity of Col-I and Col-IV substrates to support adhesion in long-term cultures could be due to reported interactions between Col-IV and laminin, a critical component of basement membranes [30]. Our preliminary data suggest that Col-IV coating might facilitate adhesion by capturing laminin that is abundantly secreted by ES cells upon LIF withdrawal and thereby promote the assembly of cell derived laminin *in vitro* ([24] and data not shown).

The important role for laminin substrate for mouse ES cell self-renewal is supported by a study from Domogatskaya et al. where LN-511 and LN-332 (both of which supported robust ES cell adhesion) were found to allow long-term self-renewal of ES cell cultures in the absence of LIF [6]. In the current study we found that, in addition to LN-511, also LN-111, FN and Col-IV enabled sustained ES cell cultures in the absence of LIF. While FN was not studied by Domogatskaya *et al.*, only limited growth of ES cell on LN-111 substrate was observed [6]. The reasons for these differences are not clear but one possibility could be the reported variability between the commercial preparations of LN-111 from Engelbreth-Holm-Swarm (EHS) sarcoma used in these studies [31,32]. However, both studies, together with other reports, confirm a positive correlation between integrin-mediated adhesion and ES cell self-renewal [6,8,10,12,13,27].

The functions of specific integrins in ES cells are not well understood but several studies report high levels of α6β1-integrin expression and/or a functional role for this integrin in ES cell adhesion [7,12,33,34]. Furthermore, αV-integrins are thought to contribute to the regulation of ES cell self-renewal [6,10,13]. Here, by using RNAi experiments we showed that β1-, α6- and αV-integrin subunits are involved in maintaining ES cell survival and self-renewal. In the absence of LIF, Itgβ1- and Itgα6-KD cells did not survive on Col I substrate as the cultures were lost prior the first passage. Importantly, depletion of α6- and of β1-integrins also affected the maintenance of stem cell marker expression profile even when LIF was present, indicating that α6β1-integrin is likely the key integrin heterodimer regulating ES cell self-renewal and pluripotency. Since α6β1-integrin is a laminin receptor, it is possible that the role of α6β1-integrin on collagen substrate is to assemble endogenous laminin secreted by ES cells themselves. Such a mechanism would be in line with the known importance of laminin during early development and maintenance of pluripotent stem cell populations [6,8,24]. Expression of αV- and β1-integrin subunits was required for the maintenance of long-term cultures on LN-511. Despite initial adhesion of individual ItgαV- and Itgβ1-KD cells to LN-511 substrate, the growing cell colonies gradually detached leading to cell loss during handling of the cultures. Integrin-mediated signaling is a complex process and, in addition to biochemical cues, mechanical cues conveyed by integrins from the ECM may also contribute to the regulation of ES cell differentiation [35]. αV-integrins are required for cellular mechanotransduction and for maturation of α2β1-integrin-mediated focal adhesions [36,37]. This phenomenon could be related to the adhesion defects observed in larger ItgαV-KD ES cell colonies.

Although both α6β1- and αVβ1-integrins can bind to laminins, our findings suggest that self-renewal of ES cells can be supported also on other substrates such as FN. Our data does not exclude the possibility that laminin is still the critical mediator of self-renewal signals because of the capacity of ES cell to produce their own laminin-rich matrix. Further studies are needed to address this possibility in more detail. However, our data clearly shows that α6β1- and αV-integrins play important roles in maintaining efficient ES cell self-renewal. Refinement of the distinct roles of α6β1- and αV-integrins in stem cell-ECM communication will help us to define stem cell niches leading to intelligible strategies to design culture conditions for *in vitro* stem cell applications. Given the multiple parallels between stem cells and cancer stem cells, targeting the function of correct integrin(s) could also help in the development of novel, more effective, approaches to limit cancer stem cell self-renewal.

## Conclusions

The present study shows that acute LIF withdrawal converts multilayered ES cell colonies into epiSC-like epithelial monolayers. Upon prolonged culture these cells remain epithelial but they regain ES cell-like expression profile of central stem cell markers and self-renew in the absence of LIF, given that they maintain a proper integrin-mediated adhesion to their substrate. Laminin-binding α6β1-integrins are critical for maintaining the ES cell-like identity, whereas αV-integrins contribute to stable adhesion on LN-511.

## Methods

### Antibodies, plasmid constructs and reagents

Primary antibodies used in this study are listed in (See Additional file 2: Table S2). HRP-conjugated (anti-rabbit, anti-mouse) antibodies were from Jackson Inc. Cy3- and Dyelight488-conjugated secondary (anti-mouse, anti-rabbit, anti-goat) antibodies were from Jackson Inc. and Alexa-488-conjugated secondary antibodies were from Invitrogen. The shRNA knockdown constructs targeting integrins were from Sigma Mission (see Additional file 2: Table S3). D-MEM (31966), β-mercaptoethanol (31350–010), and Dulbecco’s PBS were from GIBCO, ES-Qualified Fetal Calf Serum (FCS) (10439–024) was from Invitrogen. Nonessential Amino Acids (M7145), LN-111 (L2020), and LN-511 (L6274) were purchased from Sigma, FN (1918FN) was from R&D Systems, Col-I (5005-B) was from Advanced Biomatrix and Col-IV (354233) was from BD Biosciences. Leukemia Inhibitory Factor (LIF; LIF2050) was from Millipore.

### Cell culture

Mouse embryonic stem cells (ES-D3; ATCC/CRL-1934; [38]) were cultured on ECM-coated tissue culture plates in D-MEM glutamax (High Glucose, GlutaMAX™, Pyruvate) supplemented with 15% FCS (ES-Qualified), 1% Non-Essential Amino acid, 0.1 mM β-mercaptoethanol and 1% penicillin/streptomycin, at 37°C and 5% CO_2_. When indicated, 10 ng/ml LIF was added. Cell culture plates were coated by incubating plates with ECM proteins LN-111 (20 μg/ml), LN-511 (5 μg/ml), FN (10 μg/ml) in PBS, Col-I (150 μg/ml) in 0.1 M NaHCO3 buffer pH8.3 and Col-IV (10 μg/ml) in 50 mM HCl for 2 hours in +37°C and 5% CO_2_. Cells were subcultured every 4–6 days with 0.05% trypsin-EDTA (25300–054, Gibco) solution.

### Adherence assay

Cells were detached from the culture dishes using 0.05% trypsin-EDTA and counted. One thousand cells/mm^2^ were seeded onto 6-well plates coated with LN-511, Col-I or FN as indicated above in the presence or absence of 10 ng/ml of LIF. Plates were then placed into OKOLab Basic WJ CO_2_ microscope stage incubator (OKOlab) adjusted to 37°C and 5% CO_2_ and imaged at 2 min intervals for a total period of 2 hours using Olympus Cell^P live-cell/timelapse imaging system equipped with 10x phase contrast objective (Olympus). Due to plate handling, stage positioning and focusing steps, imaging was started 15–20 minutes after seeding. The first 15 frames (30 minutes) of the timelapse sequences were analysed using MTrackJ plugin for ImageJ [39]. At least fifty cells in every sequence were tracked. The cells having a total track length of less than 15 μm were considered as adhered.

### Lentiviral RNA interference

Third-generation replication incompetent lentivirus vectors were generated using a four plasmid system in HEK 293 T cells. The packaging vector containing the desired shRNA construct was from SIGMA. The helper plasmids pMD2.G (Plasmid 12259), and pMDL g/p RRE (Plasmid 12251) and pRSV-Rev (Plasmid 12253) were from Addgene [40]. DNA transfection was done using Lipofectamine 2000 from Invitrogen (11668–019). The packaging vector: pVSVG : pMDL g/p RRE : pRSV-Rev were used in 3:1:1:1 proportion and the total DNA used for transfection was 20 μg. Nearly confluent (70–80%) 293 T cells were grown on Corning CellBind (#3296) 10 cm dish. One hour before transfection, 5 ml of fresh pre-warmed medium (DMEM, low glucose, 10% FCS, 0.1% penicillin/streptomycin) was added to packing cells. To prepare transfection mix, 80 μl of Lipofectamine 2000 was added to 1 ml of OptiMEM and incubated at room temperature for 5 minutes. In another tube, 20 μg of DNA was added in the proportion indicated above to 1 ml of OptiMEM. The DNA solution was mixed with OptiMEM containing Lipofectamine drop by drop with gentle tapping of the tube. The mixture was incubated at room temperature for 20 minutes. The mixture was added on top of the cells, swirled gently and cells were placed into humidified incubator (+37°C, 5% CO_2)_. 24 hours post transfection, media containing the transfection mix was removed carefully and 5 ml of fresh media was added. The media supernatant containing the viral particles was collected every 12 hours for 3–4 days and stored at +4°C. The viral supernatants were pooled and centrifuged at 1000 rpm for 5 minutes and filtered through a 0.44 μm filter. The filtered viral supernatant was then concentrated by ultracentrifugation at 100000 X g for 2 hours. The supernatant was discarded and the viral pellet resuspended in complete DMEM (1/100th of original volume).

The cells were infected using 100 X concentrated virus particles for 24 hours in the presence of 4 μg/ml polybrene (107689, Sigma-Aldrich) and the infected cells were selected using puromycin (4 μg/ml, Sigma). The puromycin selected cells were expanded and stored by freezing in 90% ES-Qualified FCS, 10% DMSO (Sigma) in liquid nitrogen. Integrin β1-knockdown cells did not revive upon freezing, so the Itgβ1-KD cell lines were freshly made for every analysis.

### Quantitative PCR

Total RNA was isolated from ES cells cultured on different ECM coating conditions using Qiagen RNA easy column following manufacturer’s instructions. The isolated total RNA was DNase-treated (Fermentas) followed by inactivation of the enzyme by incubating the samples at 65°C for 20 minutes. 1 μg total RNA was annealed with 25 μg/ml oligo-(dT) at 70°C for 10 minutes and chilled on ice for 2 minutes. cDNA synthesis was done using 50 mM TrisCl pH 8.3, 50 mM KCl, 4 mM MgCl_2_, 10 mM DTT, 0.5 mM of each dNTP and 200 U/ml M-MLV reverse transcriptase at 42°C for 1 hour and 70°C for 15 minutes. The synthesized cDNA was then diluted 10-fold and used as a template for qRT-PCR. qRT-PCR was performed using Brilliant III Ultra-FAST SYBR Green qPCR master mix (Stratagene). The primers used in this study are listed in (See Additional file 2: Table S3). Briefly, 0.3 μM of each primer was mixed with 2 μl of diluted cDNA, 1 X SYBR and the final volume made up to 10 μl. The reactions were carried out at 1 cycle of 95°C 3 min, 40 cycles of 95°C 20 sec and 65°C 20 sec and 1 cycle at 95°C 1 min 55°C 30 sec and 95°C 30 sec (MX3005P, Stratagene). The analysis of the data was done in Microsoft Excel.

### Immunofluorescence microscopy

ES-D3 cells were cultured in the 24-well plates containing coverslips coated with the indicated ECM molecules as described above. Cells were washed with PBS, fixed in 4% PFA for 10 minutes at room temperature or at 4°C overnight. Cells were permeabilized and non-specific binding sites were blocked with PBS-Glycine (20 mM) containing 1% BSA and 0.1% Triton X-100 in for 20 minutes at room temperature. Cells were washed once with PBS containing 0.5% BSA and 0.2% glycine and incubated with the indicated primary antibodies overnight at 4°C. Subsequently the coverslips were washed 3 times and incubated with secondary antibody in PBS containing 0.5% BSA and 0.2% glycine on an ice bath for 1–2 hours in dark. The cells were washed and mounted using Immu-Mount™ (ThermoScientific). The slides were then analyzed using Olympus FluoView FV1000 confocal microscope.

### Cell doubling rate analysis

ES-D3 cells were passaged for a period of 2 months in the indicated substrate coated dishes. The cells were seeded at a density of 300 cells/mm^2^. Cell numbers were counted at the end of every passage. Cell doubling rate represents the average number of cell divisions of a single cell in 24 hours. Cell doubling is calculated using the online cell doubling calculator [41].

### Western blotting

The cells cultured on different ECM coatings, as described above, were collected and lysed using RIPA lysis buffer (10 mM Tris–HCl pH7.5, 0.5% SDS, 1% IGEPAL, 0.15 M NaCl, 1% Sodium deoxycholate + protease and phosphatase inhibitors), sonicated for 3 minutes at 100% amplitude in a QSonica800 water bath sonicater. The sonicated lysates were then centrifuged to remove insoluble material. Protein concentration was estimated using the BCA method (23225; Pierce). 10–30 μg of total protein was loaded onto a 12% gels for SDS electrophoresis, and the proteins were transferred to polyvinylidene difluoride membranes. Membranes were blocked with 5% BSA in TBS-0.1% Tween buffer for 2 hours. Primary antibodies were diluted into TBS-0.1% Tween buffer containing 1% BSA and incubated with the membranes overnight at +4°C. Membranes were washed three times, followed by addition of HRP-conjugated secondary antibodies (1:5000) in TBS-0.1% Tween buffer containing 1% BSA and incubation for 2 hours at room temperature. The membranes were washed 3–5 times in TBS-0.1% Tween buffer followed by detection of proteins by chemiluminescence using LAS-3000 imaging system (Fujifilm).

## Additional files

Additional file 1:
**Movie S1.** Examples of cell adherence assay using timelapse microscopy and cell tracking. ES-D3 cells were seeded onto 6-well tissue culture plates coated with LN-511 in the presence (upper left panel) or absence of 10 μg/ml LIF (upper right panel) or onto Col-I (lower left panel) or FN (lower right panel) in the absence of LIF. Cells were imaged at 2 min intervals for 30 minutes and cell tracks were determined. Cell adherence was determined from these data as described in materials and methods (Figure 1C, D). Additional file 2: Table S1. Integrin expression in murine ES cells. **Table S2.** Primary antibodies used in this study. Table S3. Oligos used in this study. Additional file 3: Figure S1. Expression of self-renewal and differentiation markers in control and in Itgα6-, ItgαV- and Itgβ1-KD cells. Control, Itgα6-, ItgαV- and Itgβ1-KD ES-D3 cells were grown on LN-511-, Col-I- and FN-coated tissue culture plates in the absence of LIF, total RNA was extracted at day 5, passage 3 (P3) and P5 and the mRNA expression levels of self-renewal markers (Nanog, Sox2, and Oct3/4), ES cell markers (Rex1, Klf4 and Tbx3) and a differentiation marker (FGF5) were analyzed by qPCR. The respective mRNA expression levels were compared relative to levels obtained in ES-D3 cells grown on LN-511 in the presence of LIF on day 5. The raw data values of means +/−STD are shown. The graph represents data from 2 independent experiments performed in duplicates. P-values < 0.005 are depicted with an asterisk (*). Cross (†) indicates samples where sufficient amount of cells could not be harvested for analysis. Additional file 4: Figure S2. ES-D3 cells have epithelial morphology in long-term cultures. A) ES-D3 cells (300 cells/mm^2^) were seeded onto LN-511-, Col-I- or FN-coated tissue culture dishes (3.5 cm Ø) and cultured in the absence or presence of 10 ng/ml of LIF. Upon confluency the cells were trypsinized, counted and reseeded at 300 cells/mm^2^. After 5 passages in culture (~one month) cells were imaged using a phase contrast microscope equipped with a CCD-camera. B) ES-D3 cells were cultured as in A), washed twice with PBS, fixed with 4% PFA and stained for an epithelial AJ marker E-Cad (red) and a TJ marker ZO-1 (green). Cells were imaged using confocal microscopy at the level of TJs. Scale bar is 50 μm.

## Abbreviations

ES cells: Embryonic stem cells
ECM: Extracellular matrix
EpiSCs: Epistem cells
LIF: Leukemia inhibitory factor
RNAi: RNA interference
Col: Collagen
LN: Laminin
FN: Fibronectin
Itg: Integrin
KD: Knockdown
qPCR: Quantitative polymerase chain reaction
