# Supplementary material for: α6β1- and αV-integrins are required for long-term self-renewal of murine embryonic stem cells in the absence of LIF
Source: BMC Cell Biol. 2015 Feb 27;16:3. doi: 10.1186/s12860-015-0051-y (PMC4348401; doi:10.1186/s12860-015-0051-y)
Supplement: Additional file 2: Table S1. — Integrin expression in murine ES cells. Table S2. Primary antibodies used in this study. Table S3. Oligos used in this study. [file 12860_2015_51_MOESM2_ESM.docx]

**Supplementary table S1. Integrin expression in murine ES cells**

| **Integrin subunit** | **mRNA** | **Protein** |
| --- | --- | --- |
| α1 | negative: Affymetrix array [1]  minimal: quantitative PCR (qPCR) [2]  low: qPCR [1-3] |  |
| α2 | negative: Affymetrix array, surface probing with antibody [1]  negative: quantitative PCR (qPCR) [2]  very low but detectable: qPCR [1-3] |  |
| α3 | positive: Affymetrix array but surface-probing with antibody was negative [1]  minimal: qPCR [2]  low: qPCR [1-3] |  |
| α4 | negative: Affymetrix array [1]  low: qPCR  minimal: qPCR [3] |  |
| α5 | positive: Affymetrix array [1]  low: qPCR [3] | positive: Western Blot [2]  negative: Surface probing with antibody [1, 3] |
| α6 | positive: Affymetrix array [1]  positive: qPCR [3] | low: Western Blot [2]  positive: Surface probing with antibody [1] |
| α7 | negative: Affymetrix array [1]  minimal: qPCR [2]  positive: qPCR [3] |  |
| α8 | positive: Affymetrix array [1]  very low but detectable: qPCR [3]  minimal: qPCR [2] | negative: Western Blot [2] |
| α9 | positive: Affymetrix array [1]  low: qPCR [2, 3] | low: Western Blot [2, 3] |
| α10 | negative: Affymetrix array [1]  very low but detectable: qPCR [2, 3] |  |
| α11 | very low but detectable: qPCR [2, 3] |  |
| αV | positive: Affymetrix array [1]  positive: qPCR [3]  low: qPCR [2] | minimal: Western Blot [2]  positive: Surface probing with antibody [1] |
| αIIb | positive: Affymetrix array [1] |  |
| αL | minimal: qPCR [2] |  |
| αM | minimal: qPCR [2] |  |
| αX | negative: qPCR [2] |  |
| αD | negative: qPCR [2] |  |
| αE | positive: Affymetrix array [1] |  |
| β1 | positive: Affymetrix array[1]  positive: qPCR [2, 3] | positive: Western Blot [2]  positive: Surface probing with antibody [1] |
| β2 | positive: Affymetrix array [1]  negative: qPCR [2] | negative: Surface probing with antibody [1] |
| β3 | positive: Affymetrix array [1]  minimal: qPCR [2] | negative: Surface probing with antibody [1] |
| β4 | negative: Affymetrix array [1]  minimal: qPCR [2] | negative: Surface probing with antibody [1] |
| β5 | positive: Affymetrix array [1]  low: qPCR [2] | low: Western Blot [2] |
| β6 | negative: qPCR [2] |  |
| β7 | positive: Affymetrix array [1]  low: qPCR [2] |  |
| β8 | positive: Affymetrix array [1]  very low but detectable: qPCR [3]  negative: qPCR [2] |  |

**Supplementary table S2. Primary antibodies used in this study**

| **Name** | **Dilution** | **Application** | **Source/Catalogue no.** |
| --- | --- | --- | --- |
| Mouse Nanog, Rabbit Polyclonal | 1:1000 | Western Blotting | Abcam/ab80892 |
| Human SOX2, Rabbit Polyclonal | 1:4000 | Western Blotting | Abcam/ab97959 |
| Mouse Oct-3/4, Mouse Monoclonal | 1:1000 | Western Blotting | BD Transduction Laboratories™/611203 |
| Mouse Stella, Rabbit Polyclonal | 1:3000 | Western Blotting | Abcam/ab19878 |
| Human αV-integrin, Rabbit Polyclonal | 1:2000 | Western Blotting | Chemicon/AB1930 |
| Human α6A-integrin, Mouse Monoclonal | 1:200 | Western Blotting | Chemicon/MAB1356 |
| Human CD29, Mouse Monoclonal | 1:1000 | Western Blotting | BD Transduction Laboratories™/610467 |
| Human E-Cadherin, Mouse Monoclonal | 1:200 | Immunofluorescence | BD Transduction Laboratories™/610182 |
| Human ZO-1, Rabbit Polyclonal | 1:100 | Immunofluorescence | Zymed/61-7300 |

**Supplementary table S3. Oligos used in this study**

| **Oligo** | **Sequence** |
| --- | --- |
| Nanog | Forward: 5’-aagcctttccatgtggggca-3’  Reverse: 5-atggagcggagcagcattcc-3’ |
| Sox2 | Forward: 5’-cacaactcggagatcagcaa-3’  Reverse: 5’-tctcggtctcggacaaaagt-3’ |
| Oct3/4 | Forward: 5’-ttctgcggagggatggcata-3’  Reverse: 5’-tttccactcgtgctcctgcc-3’ |
| Rex1 | Forward: 5’- ttcaacttgcgcacccacat-3’  Reverse: 5’-cctgcctttgcgtgggttag-3’ |
| Tbx3 | Forward: 5’-cagctcacactgcagtccat-3’  Reverse: 5’-tggaggactcatccgaagtc-3’ |
| Klf4 | Forward: 5’-gcagtcacaagtcccctctc-3’  Reverse: 5’-gaccttcttcccctctttgg-3’ |
| FGF5 | Forward: 5’ -gctgtgtctcaggggattgt-3’  Reverse: 5’-cactctcggcctgtcttttc-3’ |
| GAPDH | Forward: 5’-acccagaagactgtggatgg-3’  Reverse: 5’-cacattgggggtaggaacac-3’ |
| Itgα6-KD shRNA#1 TRCN0000066148 | 5’-ccggcgtctgataaagagaggcttactcgagtaagcctctctttatcagacgtttttg-3’ |
| Itgα6-KD shRNA#2 TRCN0000066150 | 5’-ccggcggcacagcaaccttgaatatctcgagatattcaaggttgctgtgccgtttttg-3’ |
| ItgαV-KD shRNA#1 TRCN0000066589 | 5’-ccggcgagggaagttacttcggattctcgagaatccgaagtaacttccctcgtttttg-3’ |
| ItgαV-KD shRNA#2 TRCN0000066591 | 5’-ccgggccagcccattgagtttgattctcgagaatcaaactcaatgggctggctttttg-3’ |
| Itgβ1-KD shRNA#1 TRCN0000066645 | 5’-ccgggccattactatgattatccttctcgagaaggataatcatagtaatggctttttg-3’ |
| Itgβ1-KD shRNA#2 TRCN0000066646 | 5’-ccggcccgacatcatcccaattgtactcgagtacaattgggatgatgtcgggtttttg-3’ |

**Supplementary references**

1. Domogatskaya A, Rodin S, Boutaud A, Tryggvason K: **Laminin-511, but not -332, -111 or -411 enables mouse embryonic stem cell self-renewal in vitro.** Stem Cells 2008, **26**(11):2800-2809.

2. Lee ST, Yun JI, Jo YS, Mochizuki M, van der Vlies AJ, Kontos S, Ihm JE, Lim JM, Hubbell JA: **Engineering integrin signaling for promoting embryonic stem cell self-renewal in a precisely defined niche.** Biomaterials 2010, **31**(6):1219-1226.

3. Hayashi Y, Furue MK, Okamoto T, Ohnuma K, Myoishi Y, Fukuhara Y, Abe T, Sato JD, Hata R, Asashima M: **Integrins regulate mouse embryonic stem cell self-renewal.** Stem Cells 2007, **25**(12):3005-3015.
