# Supplementary figures and images for: α6β1- and αV-integrins are required for long-term self-renewal of murine embryonic stem cells in the absence of LIF
Source: BMC Cell Biol. 2015 Feb 27;16:3. doi: 10.1186/s12860-015-0051-y (PMC4348401; doi:10.1186/s12860-015-0051-y)

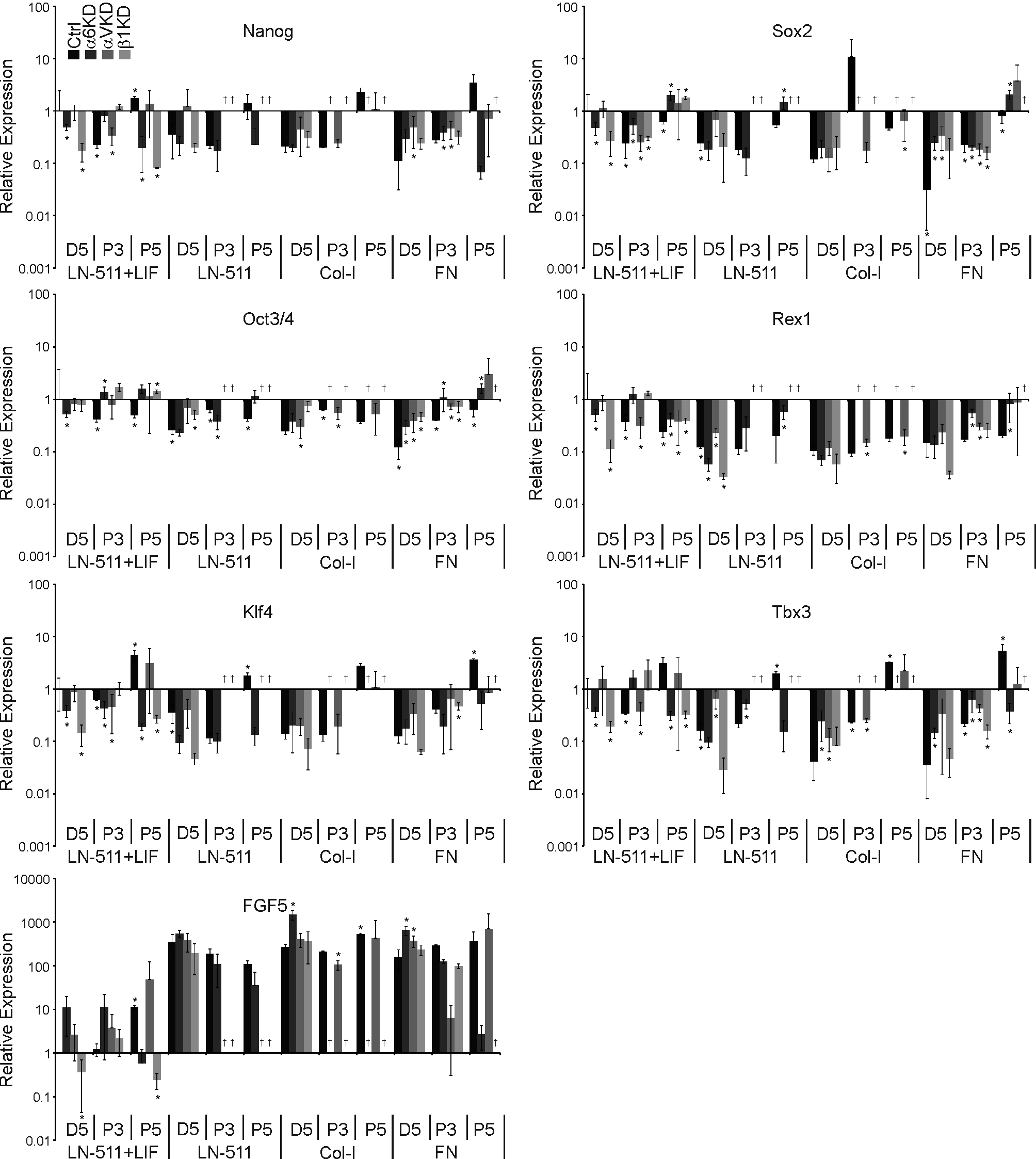

Supplement: Additional file 3: Figure S1. — Expression of self-renewal and differentiation markers in control and in Itgα6-, ItgαV- and Itgβ1-KD cells. Control, Itgα6-, ItgαV- and Itgβ1-KD ES-D3 cells were grown on LN-511-, Col-I- and FN-coated tissue culture plates in the absence of LIF, total RNA was extracted at day 5, passage 3 (P3) and P5 and the mRNA expression levels of self-renewal markers (Nanog, Sox2, and Oct3/4), ES cell markers (Rex1, Klf4 and Tbx3) and a differentiation marker (FGF5) were analyzed by qPCR. The respective mRNA expression levels were compared relative to levels obtained in ES-D3 cells grown on LN-511 in the presence of LIF on day 5. The raw data values of means +/−STD are shown. The graph represents data from 2 independent experiments performed in duplicates. P-values < 0.005 are depicted with an asterisk (*). Cross (†) indicates samples where sufficient amount of cells could not be harvested for analysis. [file 12860_2015_51_MOESM3_ESM.tiff]

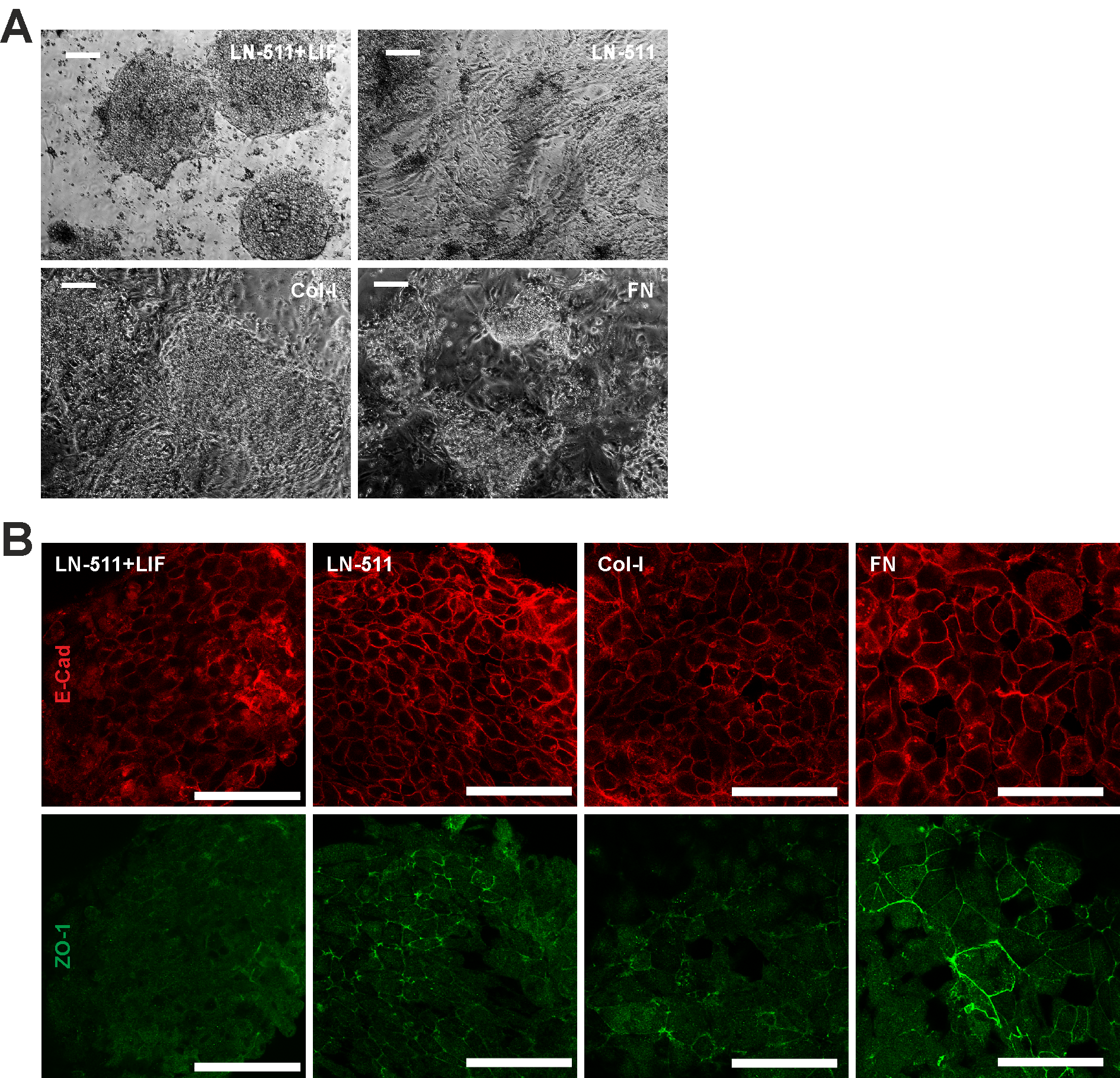

Supplement: Additional file 4: Figure S2. — ES-D3 cells have epithelial morphology in long-term cultures. A) ES-D3 cells (300 cells/mm2) were seeded onto LN-511-, Col-I- or FN-coated tissue culture dishes (3.5 cm Ø) and cultured in the absence or presence of 10 ng/ml of LIF. Upon confluency the cells were trypsinized, counted and reseeded at 300 cells/mm2. After 5 passages in culture (~one month) cells were imaged using a phase contrast microscope equipped with a CCD-camera. B) ES-D3 cells were cultured as in A), washed twice with PBS, fixed with 4% PFA and stained for an epithelial AJ marker E-Cad (red) and a TJ marker ZO-1 (green). Cells were imaged using confocal microscopy at the level of TJs. Scale bar is 50 μm. [file 12860_2015_51_MOESM4_ESM.tiff]
